# Higher Prevalence of Epstein–Barr Virus DNA in Deeper Periodontal Pockets of Chronic Periodontitis in Japanese Patients

**DOI:** 10.1371/journal.pone.0071990

**Published:** 2013-08-26

**Authors:** Ayako Kato, Kenichi Imai, Kuniyasu Ochiai, Yorimasa Ogata

**Affiliations:** 1 Department of Periodontology, Nihon University School of Dentistry at Matsudo, Chiba, Japan; 2 Department of Microbiology, Division of Immunology and Pathobiology, Dental Research Center, Nihon University School of Dentistry, Tokyo, Japan; 3 Research Institute of Oral Science, Nihon University School of Dentistry at Matsudo, Chiba, Japan; The University of North Carolina at Chapel Hill, United States of America

## Abstract

Periodontitis, a complex chronic inflammatory disease caused by subgingival infection, is among the most prevalent microbial diseases in humans. Although traditional microbiological research on periodontitis has focused on putative bacteria such as *Porphyromonas gingivalis*, the herpes virus is proposed to be involved in the pathogenesis of periodontitis because bacterial etiology alone does not adequately explain various clinical aspects. In this study, we established for the first time, more Epstein–Barr virus (EBV) DNA is found deeper in periodontal pockets of chronic periodontitis in Japanese patients. Subgingival samples were collected from 85 patients with chronic periodontitis having two periodontal sites with probing depths (PD) of ≤3 mm (shallow) or ≥5 mm (deep) and were subjected to a nested polymerase chain reaction. EBV DNA was more frequently detected in patients with deeper PD sites (66%) than in those with shallow PD sites (48%) or healthy controls (45%). Coexistence of EBV DNA and *P. gingivalis* was significantly higher in patients with deeper PD sites (40%) than in those with shallow PD sites (14%) or healthy controls (13%). Although no difference in clinical index for periodontitis, the odds ratio of EBV DNA in patients with deeper PD sites was 2.36, which was 2.07-fold higher than that in those with shallow PD sites. Interestingly, the odds of acquiring chronic periodontitis (PD ≥5 mm) were higher in the presence of both EBV DNA and *P. gingivalis* compared with either EBV DNA or *P. gingivalis* only. In addition, we also observed that EBV-encoded small RNA (EBER) in positive cells of human gingival tissues. These results would suggest that EBV DNA may serve as a pathogenic factor leading to chronic periodontitis among Japanese patients.

## Introduction

Periodontitis is a complex chronic inflammatory disease that is among the most prevalent microbial diseases in the world [1], [2]. Periodontitis affects the periodontium, and severe periodontitis can result in its destruction, occasional pain, alveolar bone resorption, and eventual tooth loss. It is now evident that host immune responses against infection with bacteria and the subsequent production of proinflammatory cytokines are of particular importance in periodontium destruction [1], [2]. Although no single etiological agent has been identified, a number of putative bacteria are considered to be associated with the disease and are used as diagnostic markers [3], [4]. *Porphyromonas gingivalis* and *Tannerella forsythia* are considered markers of adult chronic periodontitis, and *Aggregatibacter actinomycetemcomitans* is associated with aggressive periodontitis characterized by rapid alveolar bone loss [3], [4]. However, bacterial activity alone has not been able to explain the several clinical characteristics of periodontal diseases [5]. In addition, several reports have demonstrated the absence of putative periodontal bacteria in patients with periodontal disease, and there was no significant difference in the prevalence of bacteria between healthy and diseased periodontium [6]–[9]. Moreover, herpes virus has been suggested to be involved in the etiology of periodontal diseases.

Epstein–Barr virus (EBV) is an enveloped herpes virus with double-stranded DNA that infects humans only [10]. EBV is one of the most common viruses in humans, infecting more than 90% of the adult population worldwide [11], [12]. Activation of latent EBV results in viral progeny and contributes to the pathogenesis of several human diseases, including infectious mononucleosis, autoimmune disorders, and a number of malignancies [10], [13]. Although a causal relationship between periodontitis and EBV infection has not yet been established, a positive association has been reported between periodontitis and EBV infection [14], [15]. EBV is frequently found in the gingival crevicular fluid, saliva, salivary glands, and gingival tissues [16]–[19]. In addition, higher levels of EBV DNA have been detected in the saliva of patients with chronic or aggressive periodontitis [20]–[22]. Recently, we demonstrated a relationship between microbial interactions and the etiology of periodontal diseases and discovered that *P. gingivalis* can induce EBV reactivation through epigenetic regulation [23].

EBV transmission usually occurs through saliva [24], [25]; EBV DNA was detected in the throat washings of healthy adults and in the saliva of healthy children in Japan [26]. Although EBV prevalence in periodontal pockets differs with population and genetic predisposition, no studies have evaluated, as per our knowledge, EBV prevalence among of Japanese patients with chronic periodontitis. The purpose of this study was to examine whether higher prevalence of EBV DNA is associated with deeper periodontal pocket found in Japanese patients with chronic periodontitis.

## Materials and Methods

### Sampling

In this study, 20 periodontally healthy individuals (mean age, 45.9±17.0 years) and 85 chronic periodontitis patients (mean age, 57.4±13.1 years) were included. They received dental care at Nihon University Hospital School of Dentistry at Matsudo, Japan. The Institutional Internal Review and Ethics Board at the Nihon University School of Dentistry at Matsudo approved the study (EC11-027). Written informed consent was obtained from each study subject after all procedures had been fully explained. Periodontal status was assessed by probing depth (PD), clinical attachment level (CAL), and bleeding on probing (BOP). The PD and CAL were measured with a PCP11 probe (Hu-Friedy, Chicago, IL, USA). Chronic periodontitis patients were defined as having at least two sites with PD ≥5 mm and attachment loss of >5 mm. A group of 20 individuals without periodontitis were included as the healthy control group. The healthy controls showed no clinical signs of gingivitis or attachment loss, no detectable bone loss on radiographic examination, and a PD of ≤3 mm. All patients were systemically healthy and had no history of periodontal treatment or any type of antibiotic therapy for at least 3 months prior to participation in the present study. A total of 170 subgingival plaque samples were collected from two periodontal PD sites [≥5 mm (deep) and ≤3 mm (shallow)] in 85 patients with chronic periodontitis, and 40 subgingival plaque samples were collected from two PD sites (≤3 mm) in 20 periodontally healthy controls. Before sampling, the supragingival plaque was removed with sterile cotton pellets. Sterile paper points were then inserted into the sample site and retained for 30 s (three paper points used per sample site). The paper points were pooled in microcentrifuge tubes and stored at −70°C until DNA extraction.

Gingival tissues obtained during periodontal flap surgery from chronic periodontitis patients were used in this study (EC11-027).

### DNA Extraction and Polymerase Chain Reaction (PCR)

DNA extraction from the clinical samples was performed using the High Pure Viral Nucleic Acid Kit according to the user manual (Roche Applied Science, Mannheim, Germany). For the detection and typing of EBV DNA in the samples, nested PCR protocols were used, modifying those described previously for amplification of the *EBNA2* [11], [27]. DNA extracted from the cell lines Raji and AKATA were used as positive controls, and human placenta DNA was used as a negative control [26]. The first PCR amplified *EBNA2*, generating a DNA fragment of 237 bp for EBV-1 and 253 bp for EBV-2 and was performed using the following primer sets: EBV first forward, 5′-GCGGGTGGAGGGAAA GG-3′; EBV first reverse, 5′-GTC AGC CAA GGG ACG CG-3′. With second nested primers, the PCR product comprised of 168 bp for EBV-1 and 184 bp for EBV-2. The second PCR was performed using the following primer sets: EBV second forward, 5′-AGG CTG CCC ACC CTG AGG AT-3′; EBV second reverse, 5′-GCC ACC TGG CAG CCC TAA AG-3′. The amplification reactions were performed in 25 µl of final reaction mixture containing: 2× KAPA Taq Extra HotStart Ready Mix (KAPA Biosystems, Buenos Aires, Argentina); 5 µM forward and reverse primers; and 100 ng (1 µl) DNA. The thermal cycling condition (1st and 2nd PCR) was 95°C for 3 min, 35 cycles at 95°C for 15 s, 63°C for 15 s, and 72°C for 30 s, with a final extension at 72°C for 1 min. We counted EBV-1 and EBV-2 together to quantify total EBV.

For detecting *P. gingivalis,* we used PCR primers against 16S rDNA as follows, forward, 5′-TGTAGATGACTGATGGTGAAAACC-3′; and reverse primer, 5′-ACGTCATCCCCACCTTCCTC-3′ [29], [30]. The amplification reaction was the same as EBV nested PCR. The thermal cycling condition was 95°C for 3 min, 35 cycles at 95°C for 15 s, 59°C for 15 s, and 72°C for 30 s, with a final extension at 72°C for 1 min. The PCR-amplified product (*P. gingivalis*; 197 bp) was analyzed by 2% agarose gel stained with ethidium bromide upon preparation.

### Histological Examinations and In-situ Hybridization

Gingival tissues were fixed in 10% formalin solution. These specimens were embedded in paraffin and stained with hematoxylin-eosin (HE) for histological examinations. EBV was detected by in-situ hybridization (ISH) with EBV-encoded small RNA (EBER) probes. The immunohistochemical staining of CD19 (diluted at 1∶250, DAKO) was performed using streptavidine-biotine-peroxidase, and then visualized with 3,3′-diaminobenzidine trahydrochloride. The sections were then counterstained with Mayer’ hematoxylin.

### Statistical Analysis

Chi-square tests for independence testing confirmed by Fisher’s exact probability test was used to determine whether individual bacteria were associated with chronic periodontitis and to calculate the odds ratios. *P*-values of ≤0.05 were considered to be statistically significant.

## Results

The age, sex, and PD and BOP distributions of the patients are listed in Table 1. The average PD of the healthy controls (PD ≤3 mm) was 2.73±0.45 mm. In patients with chronic periodontitis (*n* = 85), the average depth of the two periodontal PD sites (≤3 mm and ≥5 mm) was 2.91±0.36 mm and 6.18±1.04 mm, respectively. The prevalence of EBV DNA in the healthy controls and patients with chronic periodontitis is listed in Table 2. The periodontopathic bacterium *P. gingivalis* was also evaluated. EBV DNA was detected in 18 (45%) periodontal pockets of healthy controls and in 41 (48%) and 56 (66%) of the shallow (≤3 mm) and deeper PD sites (≥5 mm) of patients with chronic periodontitis, respectively. No difference in EBV DNA detection rate between males and females. EBV DNA occurred at significantly higher frequencies in deeper PD sites of patients with chronic periodontitis than in PD sites of healthy controls (*P*<0.05). In addition, EBV DNA was significantly more frequent in deeper PD sites than in shallow PD sites of patients with chronic periodontitis (*P*<0.05). The occurrence frequency of *P. gingivalis* was similar to that of EBV DNA in both the healthy controls and in patients with chronic periodontitis. Coexistence of EBV DNA and *P. gingivalis* was significantly higher in the deeper PD sites of patients with chronic periodontitis (40%) than in the PD sites of the healthy controls (13%) and shallow PD sites of patients with chronic periodontitis (14%) (*P*<0.01). These results suggested that there may be a correlation between the presence of EBV DNA and a deeper PD (≥5 mm).

**Table 1:** Patient characteristics.

	HC (20 healthy controls)	CP (85 CP patients)
**Age**	45.9±17.0	57.4±13.1
**Males**	3 (15%)	36 (42%)
**Females**	17 (85%)	49 (58%)
**PD**	2.73±0.45 (n = 40)	2.91±0.36 (≤3 mm)
		6.18±1.04 (≥5 mm)
**BOP**	1 (2.5%) (n = 40)	9 (11%) (≤3 mm)
		51 (60%) (≥5 mm)

Healthy controls (HC); Chronic periodontitis (CP); PD, probing depth;

BOP, bleeding on probing.

**Table 2:** Occurrence of EBV DNA and periodontopathic bacteria in the subgingival samples from HC and patients with CP.

	Detection frequency			Significance (*P*-value)		
Microorganisms	HC	CP (≤3 mm)	CP(≥5 mm)	HC vs. CP(≤3 mm)	HC vs. CP (≥5 mm)	CP(≤3 mm) vs. CP(≥5 mm)
EBV	18 (45%)	41(48%)	56 (66%)	0.44	0.022*	0.015*
*P. gingivalis*	16 (40%)	34 (40%)	55 (65%)	0.58	0.008**	0.001**
**EBV + ** ***P.*** * gingivalis*	5 (13%)	12 (14%)	34 (40%)	0.52	0.0013**	0.0001**

Statistically significant; *P*<0.01**, *P*<0.05*; HC, healthy controls; CP, chronic periodontitis.

Clinical indices such as average PD and frequency of BOP in deeper PD sites of patients with chronic periodontitis in which EBV DNA detected alone (20 sites), *P. gingivalis* alone (19 sites), and coexistence of EBV DNA and *P. gingivalis* (36 sites) are shown in Table 3. In 10 of the deeper PD sites (total 85 sites), neither EBV DNA nor *P. gingivalis* were detected. Although the frequency of BOP in areas with EBV DNA alone (65%), *P. gingivalis* alone (58%), and EBV DNA+*P. gingivalis* (61%) was higher than in sites where these microorganisms were not detected (50%), the difference was not significant. In addition, there was no association between the average PD and the detected microorganisms in patients with chronic periodontitis (Table 3). Although no difference in clinical indices for periodontitis, the odds ratio of EBV was dependent on depth of periodontal pockets (Table 4). To calculate the odds ratios of qualitative risk factors for chronic periodontitis, the findings of periodontitis groups were compared with that of the healthy control group. In the shallow PD sites (≤3 mm) of patients with chronic periodontitis, the odds ratios for EBV DNA alone and *P. gingivalis* alone were approximately 1.0. The presence of both EBV DNA and *P. gingivalis* did not affect the odds ratios in the shallow PD sites. In contrast, in deeper PD sites (PD ≥5 mm) of patients with chronic periodontitis, the odds ratios for EBV DNA and *P. gingivalis* were 2.07 and 2.75-fold the odds ratio for shallow PD sites (≤3 mm), respectively. Interestingly, the odds of having chronic periodontitis (PD ≥5 mm) was higher (approximately 1.82-fold) in the presence of both EBV DNA and *P. gingivalis* compared with the odds associated with the solitary presence of either EBV DNA or *P. gingivalis*.

**Table 3:** Average PD and frequency of BOP in patients with chronic periodontitis with a PD of ≥5 mm.

Microorganisms	Number of sites (n = 85)	Average PD (mm)	Frequency of BOP (%)
EBV(−), *P. gingivalis.*(−)	10	5.90±0.94	50
EBV(+)	20	5.85±0.73	65
*P. gingivalis.* (+)	19	6.47±0.99	58
EBV(+), *P. gingivalis.*(+)	36	6.25±1.14	61

PD, probing depth; BOP, bleeding on probing.

**Table 4:** Association between EBV and chronic periodontitis.

	CP (PD ≤3 mm)	CP (PD ≥5 mm)
Microorganisms	odds ratio	odds ratio
EBV	1.14	2.36
*P. gingivalis*	1	2.75
EBV + *P. gingivalis*	1.15	4.67

CP, chronic periodontitis; PD, probing depth.

**Figure 1:**
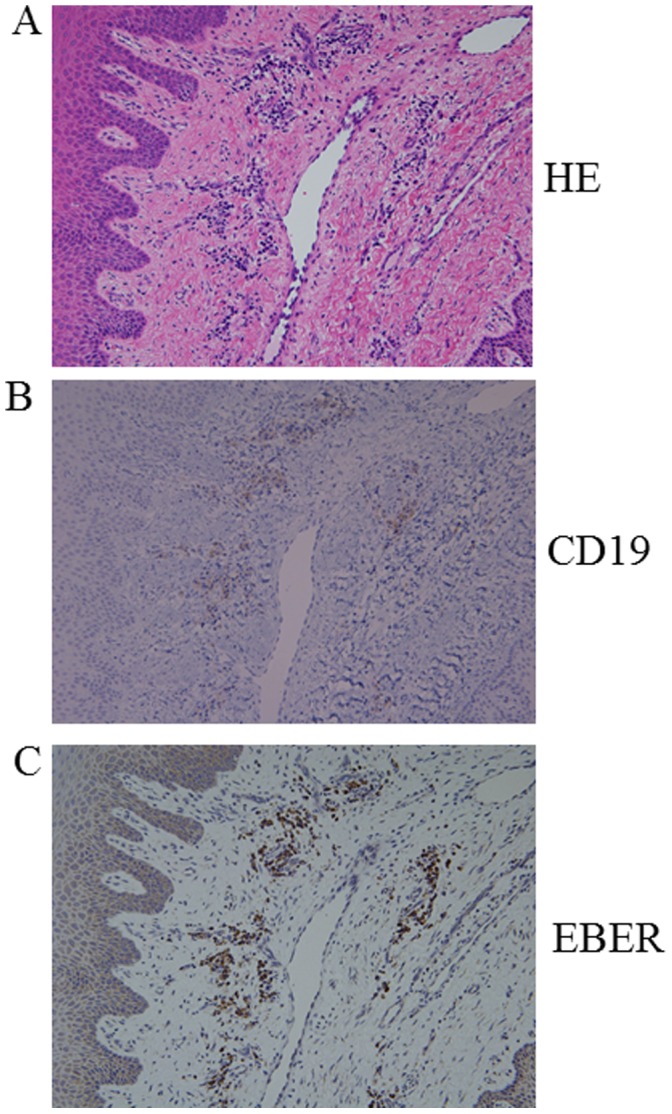
Detection of EBER in inflamed gingival connective tissue of patients with chronic periodontitis. Serial sections of periodontitis lesion were stained with HE (a), EBER ISH (b) and (c) anti CD19 antibody, respectively. Original magnification; x200.

Subsequently, we attempted to detect EBV in the gingival tissue of patients with periodontal disease in whose periodontal pockets we had previously detected EBV DNA presence. The results of B-cell marker CD19 immunostaining showed that a large number of B cells had infiltrated in the connective tissue subjacent to the gingival epithelium (Fig. 1B). Interestingly, based on the ISH results, EBV EBER showed a large number of cells in the same location that were EBER-positive (Fig. 1C).

## Discussion

Although a number of putative bacteria are considered to be associated with chronic periodontitis, it has become increasingly clear that herpes viruses are involved in the etiology of several types of periodontitis. Bacterial activity alone is not sufficient to explain the following clinical characteristics of periodontitis: rapid periodontium destruction with minimal plaque; site specificity in periodontal disease; and presence of disease activity and quiescence phases [5], [31]–[33]. In this study, we examined whether higher EBV DNA prevalence is associated with deeper periodontal pocket found in Japanese patients with chronic periodontitis. As expected, we detected EBV DNA frequently in deeper periodontal pockets.

The results of this study reveal an association between the presence of EBV DNA and chronic periodontitis lesions (PD ≥5 mm). Our results correlated with that of previous studies that showed statistically significant levels of EBV DNA in patients with chronic periodontitis compared with that in healthy controls [14], [28], [33]. Slots *et al*. discovered more EBV DNA in the gingival crevicular fluid and saliva of patients with periodontal diseases than in the saliva of an otherwise healthy control group [14], [28]. The same group demonstrated a correlation between EBV prevalence in periodontal patients and periodontal pocket depth [18], [20], [22]. Because EBV DNA detection using nested PCR is a qualitative test, we also attempted to quantitative tests using real-time PCR for some samples to support the results of nested PCR. The results showed that real-time PCR data was consistent with nested PCR data (data not shown). Real-time PCR did detect the presence of EBV DNA in those sites in which nested PCR had detected the presence of the DNA. In addition, least one order or more of EBV was detected in deeper PD sites compared with shallow PD sites (data not shown).

Although emerging evidence implicates an association between EBV and periodontal diseases, the mechanisms of EBV reactivation in the oral cavity and activated EBV progressing to periodontal disease have not yet been determined. The EBV passes through the oropharyngeal epithelium to B lymphocytes, where it establishes lifelong, latent infection [35], [36]. Reactivation can be induced *in vitro* by a variety of stimuli, including 12-*O*-tetradecanoylphorbol-13-acetate and anti-immunoglobulin [35], [36]. Because regulation of the transition from latency to reactivation is an initial key step in EBV infection, reactivation of EBV in periodontal sites is considered as an important pathogenic event in the development of periodontal disease. The results of in situ hybridization of EBV EBER showed that a large number of EBV-infected cells were observed in the connective tissue subjacent to the gingival epithelium. Countreas *et al.* reported that the types of gingival cells infected by EBV in periodontal tissue biopsy were almost CD19 positive B cells [37]. These results suggested that EBV mainly infects periodontal B lymphocyte. Recently, we demonstrated that the culture supernatant from *P. gingivalis*, which contains high concentrations of butyric acid, inhibits histone deacetylase and thus increases histone acetylation and transcriptional activity of BZLF1 gene, which encodes the master regulator protein for the transition from EBV latency to the lytic replication cycle [23]. These observations suggest that butyric acid-producing periodontopathic bacteria have the potential to trigger EBV reactivation in the oral cavity of infected patients [38].

Our research also provides evidence for potential microbial interactions between EBV and periodontopathic bacteria in the etiopathogenesis of periodontitis. Indeed, EBV and periodontopathic bacterial co-existence apparently leads to synergistic effects and exacerbates the progress of periodontal diseases [28], [34]. The EBV-infected periodontitis lesions tend to harbor elevated levels of periodontopathic anaerobic bacteria [19], [20], [28]. Furthermore, viral and bacterial co-existences were reported more frequently in deeper periodontal pockets [14], [19], [20]. Chalabi *et al.* showed that *P. gingivalis*, EBV-1, and *A. actinomycetemcomitans* were detected in 95%, 72.5%, and 12.5% of sites, respectively with probing depths of at least 6 mm [19]. We also observed that EBV DNA and *P. gingivalis* coexistence has higher detection than in areas with EBV DNA alone or *P. gingivalis* alone in deeper PD sites of patients with chronic periodontitis. In addition, an EBV DNA *–P. gingivalis* co-existence occurs in 40% of patients with chronic periodontitis (PD ≥5 mm), and the odds ratios of co-existence was the highest (4.67) compared with the odds associated with the solitary presence of either EBV DNA or *P. gingivalis*. These results support the theory that a combined presence of EBV and periodontopathic bacteria increases the risk of developing periodontitis. We assumed that the “negative chain reaction” by virus and bacteria contribute to the etiology of periodontitis. EBV infection might lead to transient local immunosuppression, thereby increasing the occurrence of periodontopathic bacteria. In turn, periodontopathic anaerobic bacteria may increase the virulence of periodontal EBV through reactivation of EBV by butyric acid.

Further studies to establish EBV as an etiologic or co-etiologic agent of periodontitis are required. New treatments and superior prevention methods can be developed with enhanced understanding of the pathogenesis of periodontitis involving EBV infections.
